# The Abuse of Salt and Vinegar

**Published:** 1918-04-06

**Authors:** G. Arbour Stephens


### THE ABUSE OF SALT AND VINEGAR.

By G. ARBOUR STEPHENS, M.D.

The sect of the Nazarites is historically very interesting, for it is one of the oldest in existence, a reference being made to it in the Pentateuch, where the vow enjoined on the members is described as including an abstinence not only from strong drink but also from vinegar. Very few people realise what a large number of diseases are aggravated, if not actually caused, by the frequent eating and drinking of acid substances, whilst in Wales one finds the inhabitants make things worse by being "heavy with the salt" as well. A large number of puny, slowly growing children are found, on inquiry, to dislike milk, but to love broths, oranges, and sour apples: if, however, one can induce the parents to be firm and stop the supply of such things, these children eventually are able to take milk and to thrive. It is a matter of everyday experience to find how difficult it is to treat medically such "acid-taking" children when they have any serious disease, and their reaction to all forms of treatment is very slow. Young women suffering from anæmia and gastric ulcer will, in the majority of cases, give a history of marked dislike to milk, but a delight in taking vinegar at every meal, even drinking it from the bottle. This class of person is very prone to suffer from chilblains, or other forms of erythema, which diseases yield to a course of calcium salts provided the diet is completely altered.

In the early summer, when the apples are small and unripe, one finds many patients complaining of urticaria, and this is traced easily to an over-indulgence in such acid fruit. Many cases of gouty eczema can be traced to a prolonged habit of drinking lemon and hot water (without the whisky) at night-time for the so-called "cure" of indigestion. It is worthy of observation that these acid-loving people are the ones who tend to develop an over-indulgence in alcohol, and the coupling of vinegar and alcohol by the ancient Nazarites is a matter of interesting and important comment. Alcoholic individuals are very fond of seasoning their food with vinegar and salt, so that we find this combination soon tends to undermine their constitutions.

As pointed out above, if these acid drinkers can be induced to drink milk, a great step forward has been made towards their reclamation. The fact that milk contains a high percentage of calcium salts is interesting, because the exhibition of calcium salts in the treatment of any diseases that these acid drinkers may suffer from is almost a *sine qua non*. Acid drinking tends to produce anæmia, as the blood, by such a habit, is deprived of much of its calcium content. At first this calcium content is kept up by calls on the other organs of the body, and these, before long, give evidence of such a drain. In rickets the percentage of calcium in the bones drops very considerably, and in other blood diseases the bones, marrow, spleen, and glands suffer (oftentimes ineffectually) on behalf of the blood system.

At the present time, when food rationing has become necessary, it is very important that the use of these condiments be reduced to a minimum, else with a lessened food supply their activities may be intensified with very evil results. If those responsible for the food supply could arrange that the people be supplied with food containing calcium salts, there need be no fear of starvation in this particular respect. It is unfortunate that at the present time there should be a scarcity of calcium salts, with a consequent marked increase in price, for, as I have pointed out on a previous occasion in The Hospital, the taking of tablets of calcium lactate is a great help to those who have to undergo any extra or heavy work, whereas, on the other hand, the poor people who never get a proper supply of calcium-containing food are very liable to suffer from ulcers of the leg.

It was in connection with the benefit produced on these chronic leg ulcers that I introduced the drug calcium iodide, and the experience has been amply confirmed by a large number of observers. The experience thus gained confirms my opinion with regard to the need for foods containing a good supply of calcium salts, and, as I have already said, the present conditions of the food supply add emphasis to this need.

